# Degradation of Waste Tetra Pak Packaging with Hydrothermal Treatment in Sub-/Supercritical Water

**DOI:** 10.3390/polym16131879

**Published:** 2024-07-01

**Authors:** Mihael Irgolič, Maja Čolnik, Petra Kotnik, Mojca Škerget

**Affiliations:** 1 Faculty of Chemistry and Chemical Engineering, University of Maribor, Smetanova ulica 17, SI-2000 Maribor, Slovenia; mihael.irgolic@um.si (M.I.); maja.colnik@um.si (M.Č.); petra.kotnik@um.si (P.K.); 2 Faculty of Medicine, University of Maribor, Taborska ulica 8, SI-2000 Maribor, Slovenia

**Keywords:** hydrothermal degradation, waste packaging, tetra pak, subcritical water, supercritical water, chemical recycling, one-stage process, two-stage process, product analysis

## Abstract

Tetra pak packaging is one of the most frequently used types of packaging in the food industry. The recycling of the tetra pak packaging waste presents a difficult task because of its multi-layered, multi-component structure. In this study, the degradation of tetra pak packaging in subcritical (SubCW) and supercritical (SCW) water was investigated. The experiments were carried out in one (SCW) or two stages (SubCW and SCW), whereby the influence of the reaction temperature and time on the yield and composition of the products obtained was investigated. The maximum oil phase yield achieved in a one-stage and a two-stage degradation process was 60.7% and 65.5%, respectively. The oil and gas phases were composed of different types of hydrocarbons. Higher temperature and longer time led to higher amounts of saturated aliphatic hydrocarbons in both the oil and gas phases. The aqueous phase contained sugars (glucose, fructose) and sugar derivatives (levulinic acid, glyceraldehyde, furfurals). Based on these results, the degradation pathway of waste tetra pak packaging in SubCW and SCW was proposed. The results of the study show that the degradation of waste tetra pak packaging with SubCW and SCW is a promising recycling process.

## 1. Introduction

The amount of packaging waste produced in the EU increased by 16.5 million tons between 2012 and 2021, while the recycling rate for packaging waste remained constant at 64–67% over the same period [1]. Almost all food is pre-packaged nowadays. One of the largest companies in the food packaging industry is Tetra Pak^®^, which produced more than 193 billion packaging units in more than 160 countries worldwide in 2022 [2]. Tetra pak packaging, which is made of cardboard (75%), aluminium (5%), and polyethylene (20%), can be used to package a variety of food products, especially fruit juices and dairy products. In the packaging composition, the cardboard, which is made from lignocellulosic material (lignin content approx. 4% [3]), provides stability and strength to the package, the aluminium acts as a barrier to air and light, while the polyethylene layer seals the contents and provides a barrier to moisture from the environment [4]. While the recycling of single-layer packaging is well researched and widely used, the recycling of multi-layer packaging represents a major problem, because its components cannot be easily separated mechanically [5].

Currently, the most common recycling method for multilayer packaging is hydro-pulping, which separates the paper portion of the packaging from the aluminium and plastic parts [5,6]. First, the waste tetra pak packaging is cut into small pieces, which are then mixed at high speed with hot water. During the mixing process, the paper fibers absorb water and form a pulp. The paper fibers pulp and Al–PE laminate are then separated by sieving [7]. The paper fibers are subsequently used to produce numerous products (tissues, paper bags). However, the remaining Al–PE composite represents a major problem for recycling. To break down the Al–PE composite, selective dissolution in various solvents (trichloroethylene, xylene, toluene, benzene) was proposed and positive separation results were obtained [8,9]. Separation of tetra pak packaging components using waste oil was also studied and gave good results for separation of the aluminium and plastic parts [10]. Pyrolysis was also used as one of the recycling techniques for waste tetra pak packaging, producing a gas phase, a waxy/oily phase, and a solid residue/ash [11]. At a temperature of 450 °C, an oil phase yield of 59.8% was reported [12].

Recently, degradation with sub- and supercritical fluids has been increasingly considered as a promising technique for the degradation of various wastes. Efforts are being made to select commonly available, harmless, and economically favorable solvents as media in which waste degradation takes place at sub- and supercritical conditions [13]. Water has been shown to be a suitable medium for the degradation of many plastic wastes (PE, PET, PS) [14,15,16] into valuable products. Water in the subcritical (SubCW) and supercritical (SCW) state enables the degradation of various wastes into useful products without the addition of other chemicals or catalysts, which reduces the negative impact on the environment [17]. The technique of hydrothermal treatment has therefore been explored to a certain extent for the degradation of multilayer packaging waste. Y. Wang et al. [18] investigated the degradation of waste tetra pak packaging in SubCW and SCW and a bio-oil yield of 25% was reported at a temperature of 360 °C, a reaction time of 30 min and a tetra pak/water ratio of 20 wt.%. Hydrothermal degradation of materials found in waste tetra pak packaging was also studied, where a positive catalytic interaction between LDPE and aluminium on the degradation of the lignocellulosic part of tetra pak packaging was found [19]. Muñoz-Batista et al. [6] found that the main solid degradation products were char and the aluminium/PE composite. The highest char content was reported at 240 °C and a time of 120 min. Lokahita et al. [20] reported that most char was obtained at 240 °C and a reaction time of 0 min, while the highest aluminium yield was found at 220 °C and a reaction time of 30 min. Additionally, Lokahita B. et al. found that the calorific value of the resulting charcoal was similar to that of lignite [21].

In this study, the feasibility of recycling and separation of multilayer packaging waste by hydrothermal treatment with SubCW and SCW is investigated. The goal was to produce products that could be used as raw materials or fuels. This study also investigated the impact of process parameters such as temperature and reaction time on the type and composition of the resulting products. The experiments were conducted with both SubCW and SCW treatments in one and two stages. The process parameters for degradation were selected based on our previous research on the degradation of cellulose [22] and PE [23] in SubCW and SCW. The products obtained from these treatments were analyzed and compared to determine the most effective approach. Additionally, the study proposes a possible pathway for the degradation of multilayer packaging waste in SCW. This pathway could provide insight into the optimal conditions for the treatment process and improve its efficiency. Overall, this research highlights the potential of hydrothermal treatment as a sustainable solution for the recycling and separation of multilayer packaging waste. The results indicate that by optimizing the process parameters, this method can produce valuable products from packaging waste, reducing its environmental impact and promoting a circular economy.

## 2. Materials and Methods

### 2.1. Material and Chemicals

Post-consumer multilayer packaging (1 L carton of milk) was collected and used as feedstock, representing waste tetra pak packaging. Standards for glucose (≥99.5%), fructose (99%), 1,6-anhydroglucose (99%), cellobiose (≥98%), glyceraldehyde (≥90%), levulinic acid (98%), 5- hydroxymethylfurfural (≥99%), furfural (99%), and 5-methylfurfural (≥98%) were supplied by Sigma-Aldrich and Merck. Dichloromethane (≥99.8%), methanol (≥99.9%), trifluoroacetic acid (99%), and sulfuric acid (99.999%) were purchased by Sigma-Aldrich. Messer (Ruše, Slovenia) supplied nitrogen (99.5%) and helium (99.999%).

### 2.2. Sample Preparation

The packaging waste was first washed thoroughly with soap and deionized water and dried at 70 °C for 24 h. Then, the packaging was cut into equal-sized pieces measuring 5 mm × 5 mm.

### 2.3. Multilayer Packaging Waste Degradation in SubCW and SCW

**Figure 1:**
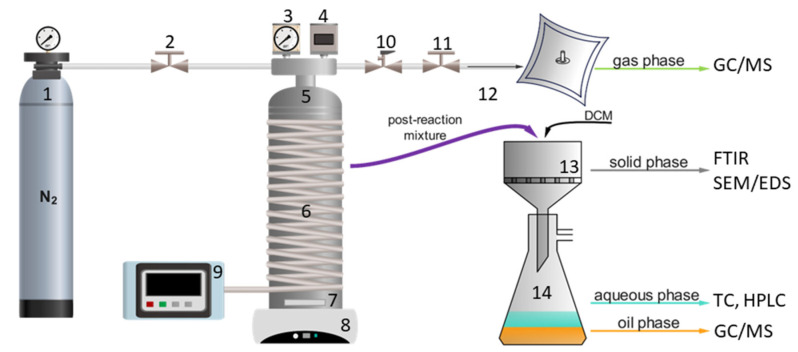
High-pressure high-temperature system. 1—N_2_ tank, 2—inlet valve, 3—pressure indicator, 4—thermocouple, 5—batch reactor, 6—heating wire, 7—magnetic stir bar, 8—electromagnetic mixer, 9—temperature controller, 10—safety valve, 11—outlet valve, 12—gas sampling bag, 13—Büchner funnel, 14—vacuum filtration flask.

SubCW and SCW degradation of multilayer packaging waste was performed in a 75 mL batch reactor with material to water ratio 1:10 The scheme of the one-stage procedure is shown in Figure 1. The detailed procedure was already described in our previous work [23], where slight modification for two-stage degradation was made. In the two-stage degradation, the solid residue on the filter paper obtained after the 1st stage was not washed with DCM but was returned to the reactor with 20 mL of fresh deionized water, and the 2nd stage experiment was performed. After the 2nd reaction stage, the phase separation was carried out as in the one-stage degradation.

A one-stage degradation was conducted in SCW at two different temperatures (425 and 450 °C) and two different reaction times (15 and 60 min). The two-stage hydrothermal degradation was carried out in the 1st stage in SubCW at a temperature of 250 °C or 300 °C and a reaction time of 30 min or 60 min. The 2nd stage of hydrothermal degradation took place in SCW at a temperature of 425 °C or 450 °C and a reaction time of 15 min. This experimental setup allowed us to understand the influence of temperature and time on the resulting product. The aim of the two-stage degradation was to remove the lignocellulosic fraction from the tetra pak waste in the first step and to remove the PE fraction of the waste tetra pak packaging in the second step. The codes of the products (oil, gas, and water samples) obtained in the two-stage degradation process in both stages at different operating parameters are listed in Table 1.

The yields of the solid, oil, water, and gas (+ losses) phases obtained were calculated according to the following Equations (1)–(4):

γ(s) = m(s)/m(m) × 100%(1)

γ(o) = m(o)/m(m) × 100%(2)

γ(aq) = m(aq)/m(m) × 100%(3)

γ(g + l) = 100% − γ(s) − γ(o) − γ(aq)(4)

where γ(s), γ(o), γ(aq), and γ(g + l) are the yield of the solid, oil, water-soluble phase, and gas + losses, respectively. m(s), m(o), and m(aq) are the weight of the solid, oil, and water-soluble components obtained after hydrothermal degradation. m(m) is the mass of waste tetra pak packaging material introduced into the reactor. In the case of two-stage degradation, all yields were calculated based on the amount of starting material loaded in the reactor in the 1st stage.

### 2.4. Material and Product Analysis

A moisture analyzer (Mettler Toledo HB43-S) was used to determine the moisture content in the feedstock material. Approximately 4 g of the sample was weighed into an aluminium pan and dried at 105 °C until constant weight. The ash content was determined by the combustion of samples at 815 °C for 3 h (standard DIN 51719) [24]. For the volatile matter determination, the crucible (with lid) is placed in the oven preheated at 900 °C (nitrogen atmosphere). After 7 min, the crucible is removed from the oven and reweighed after cooling to room temperature. The volatile matter content is calculated from the mass loss of the sample (standard DIN 51720) [24]. The content of fixed carbon was calculated from the difference [25,26]. Elemental analysis of waste tetra pak packaging was performed using a Perkin Elmer 2400 Series II System Analyzer and the content of carbon, hydrogen, nitrogen, and sulphur was determined [27]. The solid residue after degradation was analyzed by SEM/EDS (FEI SIRION 400 NC and INCA 350 EDX detector) and IRAffinity-1 FTIR spectrophotometer, using an attenuated total reflectance (ATR) cell. The spectra were recorded within the range of 4000–400 cm^−1^ against air as a background. This was achieved at a resolution of 16 cm^−1^, with a total of 20 co-added scans. Subsequently, the obtained data were analyzed using the high-performance IRsolution software. The feedstock material was subjected to thermogravimetric analysis using the Mettler Toledo TGA/DSC 1 STAR instrument. Approximately 10 mg of each sample was placed in open aluminium dishes for analysis. Measurements were performed in a nitrogen atmosphere (10 mL/min) at temperatures ranging from 25 °C to 600 °C with heating rate of 10 °C/min.

The compositions of the gas and oil phases were analyzed using the GC-MS apparatus, which consists of the Shimadzu GC-2010 gas chromatograph and the Shimadzu GC-MS 2010 QC Ultra mass selective detector. For the gas phase, the compounds were separated on an HP-PLOTQ capillary column (30 m × 0.32 mm i.d.; 20 µm film thickness) with helium serving as the carrier gas. The detailed description of the method was reported by Kotnik et al. [28]. For the oil phase, the compounds were separated using an HP-1 capillary column (30 m × 0.25 mm i.d.; 25 µm film thickness) with helium as the carrier gas (flow rate: 1 mL/min). Samples were injected in splitless mode and separation was achieved by the following temperature gradient: initially held at 40 °C for 1 min, then increased by 10 °C/min to 180 °C, further increased at 5 °C/min to 230 °C, and held at the final temperature for 5 min. The injector port and transfer line temperatures were both set to 250 °C. The mass detector conditions included electronic impact (EI) mode at 70 eV, a source temperature of 250 °C, a scanning rate of 1 scan/s, and a mass acquisition range of 35–500 m/z [29]. The identification of the compounds was based on the comparison of mass spectra with the NIST library (2014), where similarity of all compounds was more than 88%.

The total carbon (TC) in the aqueous phase was analyzed using the Shimadzu TOC-L apparatus. The concentration of TC in the aqueous phase after hydrothermal degradation of waste tetra pak packaging was calculated using the calibration curve of sodium hydrogen phthalate with a concentration range of 10 to 1 000 ppm.

The aqueous phase was analyzed using a Shimadzu Nexera HPLC system equipped with a DGU-20A SR degasser, an LC-20AD XR pump, a SIL-20AC XR autosampler, a CTO-20AC column heater, and an RI detector. A Rezex RHM-Monosaccharide H+ column with 7.8 mm i.d. and 300 mm length was used. The column temperature was set at 80 °C. The separation was achieved by an isocratic method with a flow rate of 0.6 mL/min and using 5 mM H2SO4 in water as the mobile phase. Quantification of the obtained products was performed using calibration curves of standards [22]. The analysis of furfurals (5-HMF, furfural and 5-MF) was performed by an Agilent 1100 Series HPLC system (Agilent Technologies, Waldbronn, Germany), equipped with a binary pump, an autosampler, a column heater, and a diode array detector (DAD). The column used was Agilent ZORBAX SB C18 (4.6 × 150 mm, 3.5 μm) and its temperature was 25 °C. The mobile phase A was methanol, while the mobile phase B was water + 0.1% TFA (trifluoroacetic acid). The flow rate through the column was 1 mL/min and the gradient was 0 min 90% B, 18 min 65% B, 20 min 90% B. The injection volume was 10 μL and detection of the compounds was achieved at 280 nm [30].

## 3. Results and Discussion

### 3.1. Material Characterization

The results of the proximate and ultimate analyses of the feedstock material are shown in Table 2. The volatile matter content of the waste tetra pak packaging was over 80%, suggesting that a substantial amount of gas or liquid products could be generated. The fixed carbon content was 2.8%, indicating that char could be formed during degradation. The ash content was 10.3%, suggesting that at least 10% of the waste tetra pak consists of aluminium, which could be recovered by the hydrothermal process, similar as in the pyrolysis process [31]. The high oxygen content, revealed by the ultimate analysis, can be attributed to the presence of cardboard in the material. This high oxygen content also implies that the hydrothermal degradation products could contain considerable amounts of oxygen-containing components, as predicted also by pyrolysis studies of tetra pak packaging.

**Figure 2:**
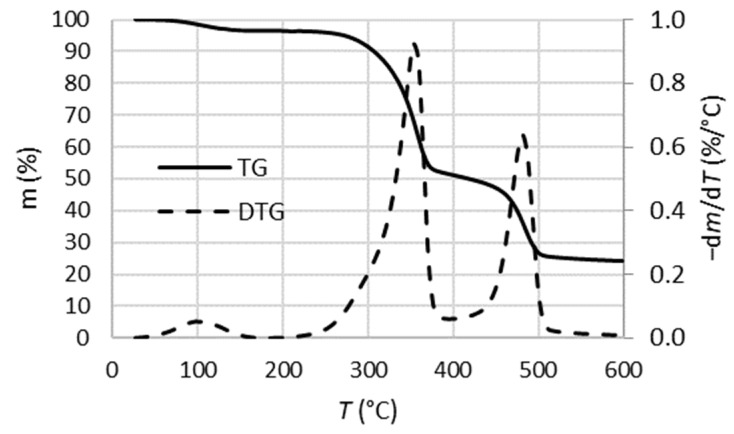
Thermogravimetric (TG) and derivative TG (DTG) curves of waste tetra pak packaging.

The decomposition profile of waste tetra pak packaging obtained with the TGA method is shown in Figure 2. After the initial mass loss (below 127 °C), which corresponds to the moisture loss, two separate sections of mass loss were clearly observed, the first for the degradation of the cardboard layer and the second for the decomposition of the plastic layer of the packaging. The first mass loss occurred between 253 °C and 371 °C, with a maximum at 354 °C, where the mass loss was 42%. The second mass loss occurred between 442 °C and 505 °C, with a maximum at 482 °C, where the mass loss was 28%. It was expected that the char is formed mainly from the cardboard part of the packaging, so the residue after 505 °C probably consisted of the aluminium part of the packaging and the char formed. The results of the analysis were consistent with previously reported studies on thermogravimetric analysis of waste tetra pak packaging [11].

### 3.2. One-Stage Degradation

**Figure 3:**
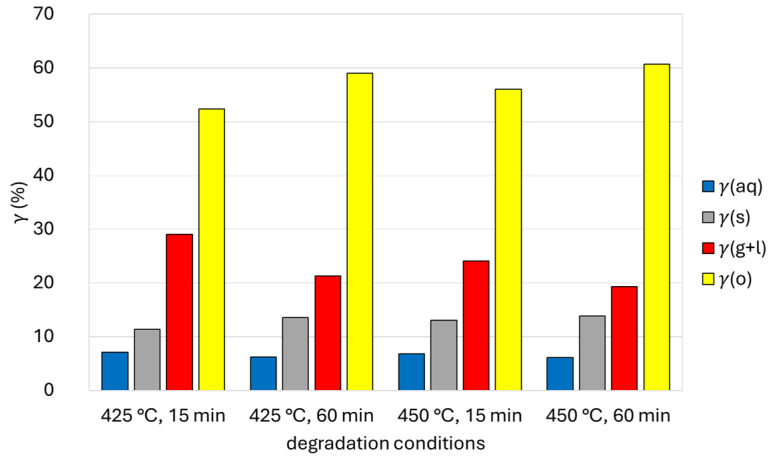
Yields of water phase *γ*(aq), oil phase *γ*(o), solid phase *γ*(s), and gas phase and losses *γ* (g + l) after one-stage hydrothermal degradation of waste tetra pak packaging.

Figure 3 shows the *γ*(aq), *γ*(o), *γ*(s), and *γ*(g + l) after one-stage hydrothermal degradation of waste tetra pak packaging. *γ*(aq) decreased from 7.1% (425 °C, 15 min) to 6.2% (450 °C, 60 min) the higher the temperature and the longer the reaction time. Conversely, the *γ*(o) increased from 52.4% (425 °C, 15 min) to 60.7% (450 °C, 60 min) with increasing temperature and reaction time. The highest *γ*(o) (60.7%) was obtained by hydrothermal degradation at a temperature of 450 °C and a reaction time of 60 min. The *γ*(o) obtained was much higher than in previous reports, where the oil yield was 20% at a temperature of 420 °C and a reaction time of 30 min [18]. It was also found that the *γ*(s) was between 11.4% and 13.9%, and increased with increasing reaction time at constant temperature, which can be the result of higher char formation at longer reaction times. The *γ*(g + l) ranged from 19.3% to 29.1% and decreased with increasing reaction time at constant temperature and with increasing temperature at constant time. The decrease in *γ*(g + l) with increasing time and temperature can be attributed to the increased formation of char. Namely, under high-temperature and high-pressure conditions (SCW), reactive gases and other products (oils, water-soluble compounds) that form simultaneously can react with each other and produce various carbon structures [32]. Light gases such as O_2_, N_2_, NH_3_, CO, CO_2_ and hydrocarbons are completely miscible in SCW, which makes SCW a good reaction medium also for homogeneous reactions of organic compounds with gases [33]. The results show that when the reaction time was increased from 15 min to 60 min, the water-soluble components were converted into oil-soluble compounds or char, which is also reflected in the reduction of the TC content of the aqueous phase. Moreover, at higher temperatures and longer reaction times, some polymerization reactions, especially from unsaturated aliphatic hydrocarbons in the presence of metal catalysts, can take place, which could explain the higher oil yields. In addition, the differences in losses may also alter the gas phase results to some extent.

#### 3.2.1. Oil Phase Composition

Due to the large number of different compounds in the oil phases determined by GC/MS (over 100), they were grouped into individual hydrocarbon groups. It was found that saturated and unsaturated aliphatic hydrocarbons (alkanes and alkenes), cyclic hydrocarbons (alicycles), aromatic compounds (arenes), alcohols, and ketones were formed during degradation. The composition of the individual oil phases after hydrothermal degradation of waste tetra pak packaging is shown in Table S1, where only those components are listed whose content in the oil is at least 1%.

Table S1 shows that the proportion of alkanes increased with increasing temperature and reaction time from 29.00% (425 °C, 15 min) to 46.33% (450 °C, 60 min). The most abundant compound among the saturated aliphatic hydrocarbons was heneicosane, which accounts for 21% to 45% of the total alkanes. Heneicosane has already been reported to be the most abundant alkane detected in the oil obtained by the degradation of PE in SCW [23]. Other commonly represented saturated aliphatic hydrocarbons determined in the oil phase were hexacosane, eicosane, nondecane, pentadecane, dodecane, and undecane. The amount of unsaturated aliphatic hydrocarbons also increased with increasing reaction time and temperature and was highest (17.15%) after degradation at 450 °C and a reaction time of 60 min. The most abundant unsaturated aliphatic hydrocarbon was 9-octadecene. A similar oil composition was reported for oil obtained by pyrolysis of tetra pak packaging at 450 °C, where the content of alkanes and alkenes was 65.1% [12]. Table S1 also shows that alicyclic hydrocarbons were present in small amounts (maximum 4.55%) or were not present at all (450 °C, 60 min), with cyclopentane derivatives being the most abundant compounds. It was observed that at a degradation temperature of 425 °C, the amount of aromatic hydrocarbons decreased with increasing reaction time (from 28.37% to 21.94%). The same is observed at 450 °C, but the total aromatic content is lower than when degrading at 425 °C. It can be concluded that at higher temperatures, the degradation of aromatic compounds into simpler compounds took place. Among the aromatic compounds, benzene and phenol derivatives were present in significant amounts. The amount of alcohols and ketones was highest after degradation at 450 °C and a reaction time of 15 min and was 16.90% for alcohols and 15.21% for ketones. It was observed that the amount of alcohols decreased with increasing reaction time, while the amount of ketones increased with increasing time at the degradation temperature of 425 °C and decreased at the degradation temperature of 450 °C.

#### 3.2.2. Gas Phase Composition

The results of the GC/MS analysis of the individual gas phases (in % peak area) after the one-step hydrothermal degradation of the waste tetra pak packaging are shown in Table 3. The gas phase consisted of CO_2_ and light saturated (ethane, propane, butane, pentane, hexane) and unsaturated (ethene, propene, 1-butene, 1-pentene) aliphatic hydrocarbons. The most common gases were CO_2_, ethane, propane and butane, which together accounted for 67% (450 °C, 15 min) to 73% (425 °C, 60 min) of the total gases in each gas phase.

It was observed that the amount of CO_2_ decreased from 36.2% (425 °C, 15 min) to 20.4% (450 °C, 60 min) as the temperature and reaction time increased. It can also be seen that, in general, the amount of unsaturated aliphatic hydrocarbons decreased with increasing degradation time at constant temperature, while, on the contrary, the amount of saturated aliphatic hydrocarbons increased. Thus, the amount of ethane increased from 14.9% to 17.5% and the amount of propane from 11.9% to 17.7% when the reaction time of hydrothermal degradation at 450 °C was prolonged, while under the same degradation conditions, the amount of ethene decreased from 2.9% to 1.4% and the amount of propene from 10.0% to 7.2%. An increase in alkane concentration and a decrease in alkene concentration with increasing reaction temperature was also observed in the degradation of HDPE in SCW [16].

#### 3.2.3. Characterization of Solid Phase

**Figure 4:**
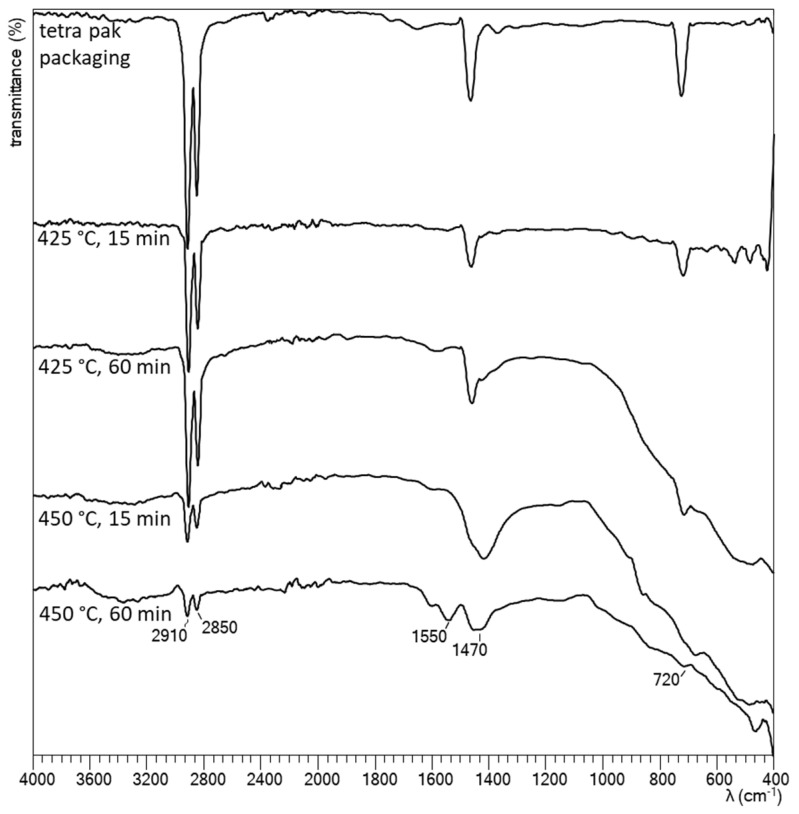
FTIR spectra of waste tetra pak packaging and of solid phase remaining after one-stage hydrothermal degradation of waste tetra pak packaging.

For the characterization of solid phase remaining after hydrothermal degradation of waste tetra pak packaging at different conditions, FTIR spectra were obtained and compared with the FTIR spectra of tetra pak packaging (shown in Figure 4). Peaks at 2910 cm^−1^ and 2850 cm^−1^ represented CH_2_ asymmetric and symmetric stretching; peak at 1470 cm^−1^ represented CH_2_ scissors and peak at 720 cm^−1^ CH_2_ rocking. Observed peaks were typical for PE and were already reported in the literature [34]. The peaks were compatible with the peaks observed for waste tetra pak packaging. When a small piece of the packaging covered with a thin PE layer was placed directly into the ATR cell, the plastic layer of the packaging was predominantly detected. To detect the metals present in the solid residue, an EDS analysis was carried out. The EDS analysis of the solid phase remained after degradation at 450 °C and reaction time of 60 min; Figure S1 and Table S2 show that the residue contains predominantly oxygen, aluminium, and silicon but there were also traces of other metals such as calcium, chromium, iron, cobalt, and nickel. These elements probably originated from the pigments in waste tetra pak packaging [35].

#### 3.2.4. Aqueous Phase Analysis

TC content in the aqueous phase after one-step hydrothermal degradation of waste tetra pak packaging (Table S3) at a reaction time of 15 min showed very similar values at both temperatures studied (3792 mg/L at a temperature of 425 °C and 3838 mg/L at a temperature of 450 °C), from which it can be concluded that at a shorter reaction time, the temperature has a lower influence on the degree of degradation. As the reaction time increased at constant temperature, the TC content in the aqueous phase generally decreased, which was more pronounced at the higher temperature of 450 °C. The highest TC value (3838 mg/L) was determined for the aqueous sample obtained at 450 °C and a reaction time of 15 min, while the lowest TC value (2466 mg/L) was determined for the aqueous sample produced at 450 °C and a reaction time of 60 min.

By the HPLC analysis of the aqueous phase formed after the one-step hydrothermal degradation of waste tetra pak packaging, glucose, glyceraldehyde, levulinic acid, 5-HMF, and furfural were detected. Table S3 shows the contents of the individual components. It was found that at both temperatures studied, the glucose concentration was significantly higher at shorter reaction times (15 min) (between 0.037 mg/mL and 0.046 mg/mL) than at longer reaction times (60 min) (from 0.002 mg/mL to 0.003 mg/mL). It can be assumed that the glucose was broken down into different derivatives with increasing reaction time. The glyceraldehyde concentration decreased with increasing temperature and reaction time from 0.077 mg/mL (425 °C, 15 min) to 0.063 mg/mL (450 °C, 60 min). The concentration of levulinic acid was highest (1.714 mg/mL) after hydrothermal degradation of waste tetra pak packaging at a temperature of 425 °C and a reaction time of 15 min and decreased with increasing reaction time. The decrease in the concentration of glyceraldehyde and levulinic acid with increasing residence time may be attributed to further degradation to gaseous products or the formation of char. 5-HMF and furfural were also detected in small amounts in the aqueous phase. The 5-HMF concentration decreased with increasing reaction time at 450 °C from 0.008 mg/mL to 0.002 mg/mL, while it was not detected at 425 °C. On the contrary, the concentration of furfural increased with increasing reaction time at constant temperature, which may be a consequence of further degradation of 5-HMF to furfural. In a study on the conversion of cellulose in SubCW and SCW, it was reported that the sugars and their derivatives are the main components obtained in the water phase [36].

### 3.3. Two-Stage Degradation

**Figure 5:**
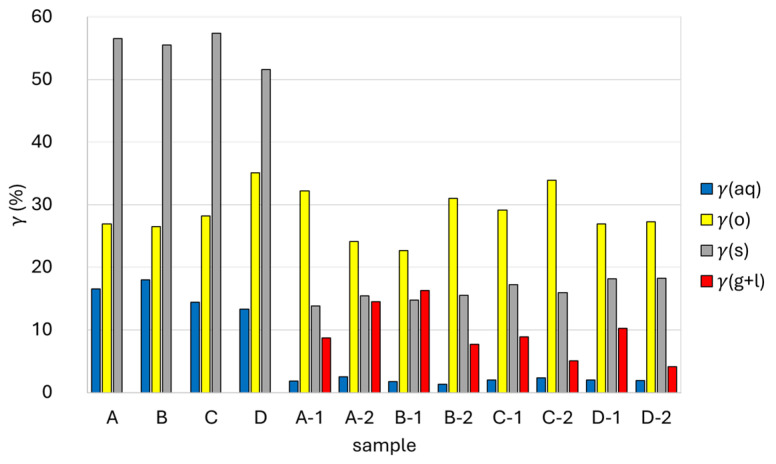
Yields of aqueous phase *γ*(aq), oil phase *γ*(o), solid phase *γ*(s), and gas phase + loss *γ*(g + l) after 1st stage and 2nd stage of two-stage hydrothermal degradation of waste tetra pak packaging.

Figure 5 shows *γ*(aq), *γ*(o), *γ*(s), and *γ*(g + l) after the 1st and 2nd stage of the two-stage hydrothermal degradation of packaging waste. The *γ*(aq) after the 1st stage was generally higher at the lower temperature studied, increased at 250 °C (from 16.5% to 18.0%), and decreased at 300 °C (from 14.4% to 13.3%) as the reaction time increased from 30 to 60 min. The highest *γ*(aq) after the 1st stage was obtained at a degradation temperature of 250 °C and a reaction time of 60 min (sample B) and amounted to 18.0%. The *γ*(o) obtained in the 1st stage at 250 °C was almost the same for both reaction times (26.9% at 30 min, 26.5% at 60 min), while it was higher at 300 °C, increasing from 28.2% to 35.1% with increasing reaction time. The *γ*(o) after the 1st stage varied between 57.4% (300 °C, 30 min, sample C) and 51.6% (300 °C, 60 min, sample D).

The products obtained in the 2nd stage of the degradation process of waste tetra pak packaging at 425 °C and a reaction time of 15 min were labeled with A-1, B-1, C-1, and D-1. The *γ*(aq) were very similar and ranged from 1.8% to 2.0%. The *γ*(o) at this temperature was the highest for sample A-1 (1st stage 250 °C, 30 min) and amounted to 32.2%. In general, it was observed that the *γ*(o) in the 2nd stage was higher for the samples degraded in the 1st stage with a shorter reaction time (30 min) than for the samples degraded with a longer reaction time (60 min), which was probably due to the higher amount of undegraded lignocellulose remaining in the solid residue after the 1st stage of degradation. The *γ*(s) ranged from 13.8% (A-1, 1st stage 250 °C, 30 min) to 18.2% (D-1, 1st stage 300 °C, 60 min). When the 2nd stage of the degradation process was carried out at 450 °C and a reaction time of 15 min (A-2, B-2, C-2, D-2), the *γ*(aq) and *γ*(s) were similar to those at 425 °C, while the *γ*(o) was generally higher, except for the experiments labeled with A, where the 1st stage was performed at 250 °C for 30 min. The *γ*(aq) ranged from 1.3% (B-2, 1st stage 250 °C, 60 min) to 2.5% (A-2, 1st stage 250 °C, 30 min), while the *γ*(s) ranged from 15.4% (A-2, 1st stage 250 °C, 30 min) to 18.3% (D-2, 1st stage 300 °C, 60 min). The highest *γ*(o) was obtained for sample C-2 (1st stage 300 °C, 30 min) and amounted to 33.9%, which was due to the fact that the largest amount of solid residue remained in the 1st stage and was further processed in the 2nd stage.

The gas phase after the 1st stage was not collected in this study. The *γ*(g + l) after the 2nd stage is represented in Figure 5. The *γ*(g + l) was between 4.2% and 16.3%. It can be observed that when the 1st stage was carried out at the lowest temperature and shortest time (250 °C and 30 min), the *γ*(g + l) obtained in the 2nd stage increased with increasing temperature of the 2nd stage (A-1, A-2). This was probably due to a larger amount of lignocellulosic material remaining after the 1st stage that was than more pronounced to the gas producing at higher temperatures in the 2nd stage. When the 1st stage is carried out at 250 °C and 60 min, the highest *γ*(g + l) was observed for sample B-1, obtained in the 2nd stage at 425 °C, and it is 16.3%. With further increase in temperature and reaction time in the 1st stage, the *γ*(g + l) obtained in the 2nd stage was generally lower and decreased with the increase in temperature of the 2nd stage from 425 °C to 450 °C (B-2, C-2, D-2), which can be due to the char formation from the remaining lignocellulosic material rather than the gas production. The *γ*(g + l) for sample D-2 (4.2%), for which it can be assumed that the solid residue after the 1st stage contained the least lignocellulosic material, was similar to that reported for the degradation of PE waste at 450 °C and 15 min [23].

#### 3.3.1. Oil Phase Composition

The oil phases obtained in the 1st and 2nd stages of the degradation process differed in color and odor. The oils obtained in the 1st stage were reddish-brown, while those obtained in the 2nd stage were dark brown (almost black) and had a more intense odor typical of gasoline.

The composition of the oil phase after the 1st and 2nd stage of the two-stage hydrothermal degradation of the waste tetra pak packaging is shown in Tables S4 and S5. In the oil phases obtained in the 1st stage, no alkanes, alkenes, alicyclic hydrocarbons, or alcohols were detected by the GC/MS analysis. The amount of aromatic compounds ranged from 27.09% to 45.18%, with the maximum being reached at 250 °C and a reaction time of 60 min (B). Among the aromatic compounds, mainly methoxy-phenol derivatives and methyl-phenol derivatives were detected, which were degradation products of lignin [18]. The ketones were mainly cyclopenten-1-one derivatives. The highest content of ketones was found in sample A (250 °C, 30 min) with 42.61% and the lowest in sample B (250 °C, 60 min) with 25.55%. It was observed that the amount of aromatic compounds increased with increasing reaction time at 250 °C, while on the contrary, the amount of ketones at 250 °C decreased with increasing reaction time.

In the oil phases obtained in the 2nd stage, the content of alkanes and alkenes was higher in the samples obtained at the higher degradation temperature (450 °C), when the reaction time in the 1st stage was 30 min. The highest content of alkanes and alkenes was found in sample C-2 (1st stage 300 °C, 30 min; 2nd stage 450 °C, 15 min) with 41.50% and 19.60%, respectively. The aliphatic hydrocarbons were represented by compounds from C_6_ to C_44_, with heneicosane and hexacosane being the most abundant compounds and eicosane, heptadecane, pentadecane, dodecane, and undecane also being detected, among others. The percentage of alicyclic hydrocarbons was low, but it was found that the content was slightly higher (between 1.80% and 5.54%) in the samples obtained in the 2nd stage at a higher temperature (450 °C) than in the samples obtained at a lower temperature (between 1.41% and 4.05%). The alicycles were mainly represented by cyclopropane derivatives and cyclohexane derivatives. The content of aromatic compounds (arenes) was higher in the samples obtained at a lower temperature (425 °C) in the 2nd degradation stage (between 9.47% and 16.88%) than in the samples obtained at a higher temperature, where the content was between 3.44% and 9.10%. The aromatic compounds were mainly various phenol- and indene-based compounds. The content of alcohols was above 22% in all samples obtained at 450 °C in the 2nd degradation stage and was highest in sample A-2 (1st stage 250 °C, 30 min; 2nd stage 450 °C, 15 min) at 31.50%. The lowest amount of alcohols was determined in sample A-1 (1st stage 250 °C, 30 min; 2nd stage 425 °C, 15 min) with only 2.35%. The alcohols were mostly linear, with heptacosanol, heneicosanol, docosanol, and heptadecanol being the most abundant. The concentration of ketones was low in all samples (below 6.5%), but it was observed that the amount in the samples where the 2nd degradation stage took place at 450 °C was lower than in the samples where the 2nd degradation stage took place at 425 °C. No ketones were detected in samples B-2 (1st stage 250 °C, 60 min; 2nd stage 450 °C, 15 min) and C-2 (1st stage 300 °C, 30 min; 2nd stage 450 °C, 15 min). The residue consisted of various esters and acids (vinyl esters, carbonic acid, etc.).

#### 3.3.2. Gas Phase Composition

GC/MS analysis results of the individual gas phases (in % peak area) obtained in the 2nd stage of the two-stage hydrothermal degradation of the waste tetra pak packaging are shown in Table 4. The gas phase consisted of CO_2_ and light saturated (ethane, propane, butane, pentane, hexane) and unsaturated (ethene, propene, 1-butene, 1-pentene) aliphatic hydrocarbons. The most common gases were CO_2_, ethane, propane, and propene.

It has been observed that the amount of CO_2_ decreased with increasing temperature in the 2nd stage. Furthermore, the amount of CO_2_ also decreased with increasing temperature and reaction time in the 1st stage. For the samples degraded at 250 °C in the 1st stage, the amount of C_5_-C_6_ hydrocarbons generally increased with increasing reaction temperature in the 2nd stage. For the samples degraded at 300 °C in the 1st stage, a decrease in the amount of C_5_-C_6_ hydrocarbons was observed with an increase in the reaction temperature in the 2nd stage. The amount of butane and propane increased with the increase of the reaction temperature in the 2nd stage and also with the reaction temperature and time in the 1st stage. Propane and butane were the main gas components (together from 15.9% to 36.8%) that were produced from the cracking of oil components. The composition of the gas phase of samples D-1 and D-2 was very similar to the composition of the gas phases obtained by degradation of PE waste [23] in SCW, indicating that a small amount of lignocellulose remained in the solid residue of the 1st stage.

#### 3.3.3. Aqueous Phase Analysis

Similar to the oils, the aqueous phases obtained in the 1st and 2nd stages of the degradation process also differed in color. The aqueous phase after the 1st stage had a yellow-brown color, while the aqueous phase after the 2nd stage was almost transparent.

**Figure 6:**
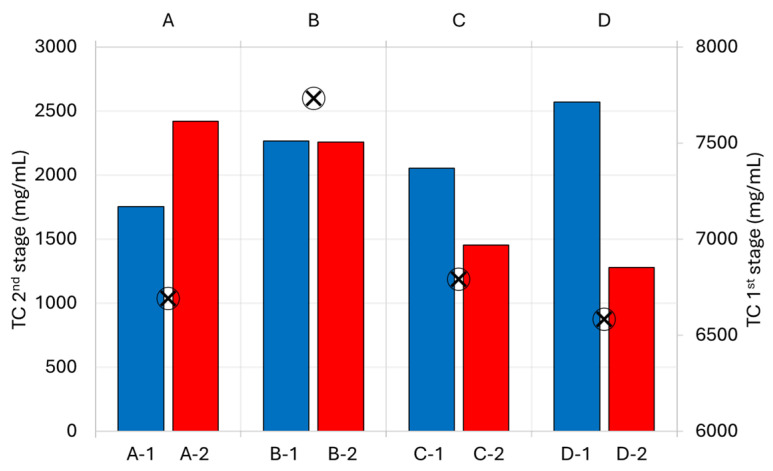
TC values in aqueous phase after 1st (symbols) and 2nd (columns) stage of two-stage hydrothermal degradation of waste tetra pak packaging.

Figure 6 shows TC concentration in the aqueous phase after the 1st (symbols) and 2nd (columns) stages of the two-stage hydrothermal degradation of waste tetra pak packaging. It was observed that the TC value in the aqueous phase at the degradation temperature of 250 °C increased from 6692 mg/L to 7733 mg/L when the reaction time was increased from 30 to 60 min. In contrast, the TC value in the aqueous phase decreased from 6791 mg/L to 6583 mg/L with increasing reaction time at a degradation temperature of 300 °C. Thus, the maximum TC value was obtained at a degradation temperature of 250 °C and a reaction time of 60 min (sample B, 7733 mg/L), at which the *γ*(aq) after the 1st degradation stage was also the highest (18.0%). For the degradation of cellulose in SubCW at 300 °C, it has been reported that the total amount of carbohydrates obtained decreased with increasing reaction time [22], hence the decrease in the TC value in the aqueous phase. It was found that the TC values after the 2nd stage were more than two times lower as the TC values after the 1st stage of degradation and were dependent on the process conditions of the 1st stage. When the 2nd stage was performed at 425 °C, the TC content in the aqueous phase increased with increasing temperature and reaction time of the 1st stage, and the highest TC content was obtained for sample D-1 (2572 mg/L). When the 2nd stage was performed at 450 °C, the TC content in the obtained aqueous phase decreased with increasing temperature and reaction time of the 1st stage and the highest TC content was obtained for sample A-2 (2422 mg/L). When the reaction temperature of the 2nd stage was increased from 425 °C to 450 °C, the water-soluble components were converted into oil-soluble compounds, which reduced the TC content.

After the 1st stage of degradation, the presence of glucose, cellobiose, glucose anhydride (1,6), levulinic acid, 5-HMF, furfural, and 5-MF was determined by HPLC analysis. Levulinic acid and glucose anhydride were the major components with concentrations ranging from 1.742 mg/mL to 2.498 mg/mL and 2.108 mg/mL to 5.410 mg/mL, respectively, while the concentrations of glucose (0.093 mg/mL–0.286 mg/mL) and cellobiose (0.013 mg/mL–0.211 mg/mL) were one or two orders of magnitude lower. The contents of each component are shown in Table S6. In the 1st stage, the concentration of glucose and levulinic acid increased with increasing temperature and reaction time, while the concentration of cellobiose decreased with increasing time at constant temperature and increased with increasing temperature at constant time. An increasing concentration of 1,6-anhydroglucose with increasing temperature and reaction time has already been reported in a study on the degradation of glucose in SubCW [37]. 5-HMF was detected at 250 °C at a concentration between 0.064 mg/mL and 0.078 mg/mL, which increased with increasing reaction time, and a small amount of furfural was detected after 60 min. At 300 °C, 5-HMF was no longer detected, but furfural and 5-MF, which are further degradation products of 5-HMF, were detected in small amounts.

After the 2nd stage of hydrothermal degradation of waste tetra pak packaging, glucose, fructose, levulinic acid, and furfurals were detected in the aqueous phase, while cellobiose and glucose anhydride were no longer present. Glucose concentration in the aqueous phase of the 2nd stage was generally lower than in the 1st stage and was significantly lower (between 0.002 mg/L and 0.039 mg/mL) in the samples where the 1st stage was performed at a degradation temperature of 250 °C (A-1, A-2, B-1, B-2) than in the samples where the 1st stage was performed at a temperature of 300 °C (C-1, C-2, D-1, D-2), where the glucose concentration ranged from 0.041 mg/mL to 0.169 mg/mL. It has been reported in the literature that the formation of glucose from cellulose when processed in SCW is significant at temperatures above 280 °C [38]. The fructose concentration was about an order of magnitude higher in the samples obtained at a 1st stage temperature of 300 °C and a 2nd stage temperature of 425 °C (samples C-1 and D-1) and were 0.293 mg/mL and 0.283 mg/mL, respectively. At a temperature of 250 °C in the 1st stage, the fructose concentration in the aqueous phase after the 2nd stage (A-1, A-2, B-1, B-2) was approximately 3.7 times higher (between 0.060 mg/mL and 0.063 mg/mL) when the reaction time of the 2nd stage was shorter (A1 and A2, 250 °C, 30 min) and was not significantly dependent on the temperature of the 2nd stage. This is because the process of isomerization of glucose to fructose took place before any other decomposition process of glucose [39]. The concentration of levulinic acid was higher in the samples where the 1st stage was carried out at a degradation temperature of 250 °C (A-1, A-2, B-1, B-2) than in the samples obtained in the 1st stage at a temperature of 300 °C (C-1, C-2, D-1, D-2). Thus, the highest levulinic acid concentration was in sample A-1 (1st stage 250 °C, 30 min; 2nd stage 425 °C, 15 min) and was 0.751 mg/mL. 5-HMF is detected only in samples A-1 (0.008 mg/mL) and A-2 (0.013 mg/mL) which were obtained at the lowest temperature and shortest time in the 1st stage and therefore more of the lignocellulosic material remained in the solid residue and the degradation in the 2nd stage was not taken further to furfural or 5-MF. The other samples of the 2nd stage contain furfural in low concentrations (from 0.004 mg/L to 0.010 mg/mL). It was observed that the concentration of furfural was higher when the 2nd stage reaction was carried out at a higher temperature. 5-MF, the further degradation product of glucose, was detected in samples D-1 and D-2, where the reaction temperature of the 1st and 2nd stage was highest and the lignocellulosic material was already highly decomposed to smaller molecules in the 1st stage.

#### 3.3.4. Solid Phase Characterization

The solid residue after the 1st stage of decomposition was in the form of pieces similar to the starting material, while the solid residue after the 2nd stage of decomposition had completely disintegrated into small particles. 

Figure S2a shows the FTIR spectra of the solid phase after the 1st stage of the two-stage hydrothermal degradation of waste tetra pak packaging. All recorded spectra showed similar peaks regardless of the reaction conditions, but they differed significantly in intensity. The peak at 3333 cm^−1^ represented the hydroxyl group (-OH), the peak at 2850 cm^−1^ represented the methyl group (-CH_2_), the peak at 1550 cm^−1^ represented the carbonyl group (C=O), the peak at 1470 cm^−1^ represented the methyl group (-CH_2_), and the peaks between 1200 cm^−1^ and 1050 cm^−1^ corresponded to the glycosidic bond (C-O-C). The recorded spectra were compared with the literature [34,40], and it was found that the peaks at 3333 cm^−1^, 1550 cm^−1^, and between 1200 cm^−1^ and 1050 cm^−1^ represented the peaks for cellulose, while the peaks at 2910 cm^−1^, 2850 cm^−1^, and 1470 cm^−1^ (and around 720 cm^−1^) were characteristic for PE. It was observed that the peaks characteristic of cellulose were of significantly higher intensity for samples obtained at a lower temperature (250 °C), indicating a higher cellulose concentration, than the peaks of samples obtained at a degradation temperature of 300 °C. This confirmed our observations when performing experiments, namely that the solid residue after the 1st stage contained more paper pulp when the degradation was performed at a lower temperature. The FTIR spectra of the solid phase recorded after the 2nd stage (Figure S2b) of the two-stage hydrothermal degradation of the waste tetra pak packaging were very similar to the spectra of the solid phase recorded after the one-stage degradation of the waste tetra pak packaging presented in Section 3.2.3. EDS analysis of the solid phase remaining after the 2nd stage of degradation (Figure S3 and Table S7) showed that the residue contained predominantly oxygen, aluminium, and silicon, but traces of other metals such as calcium, chromium, iron, cobalt, magnesium, molybdenum, and nickel were also present. These elements were also present after one-stage degradation.

### 3.4. Degradation Pathway of Waste Tetra Pak Packaging in SubCW and SCW

**Figure 7:**
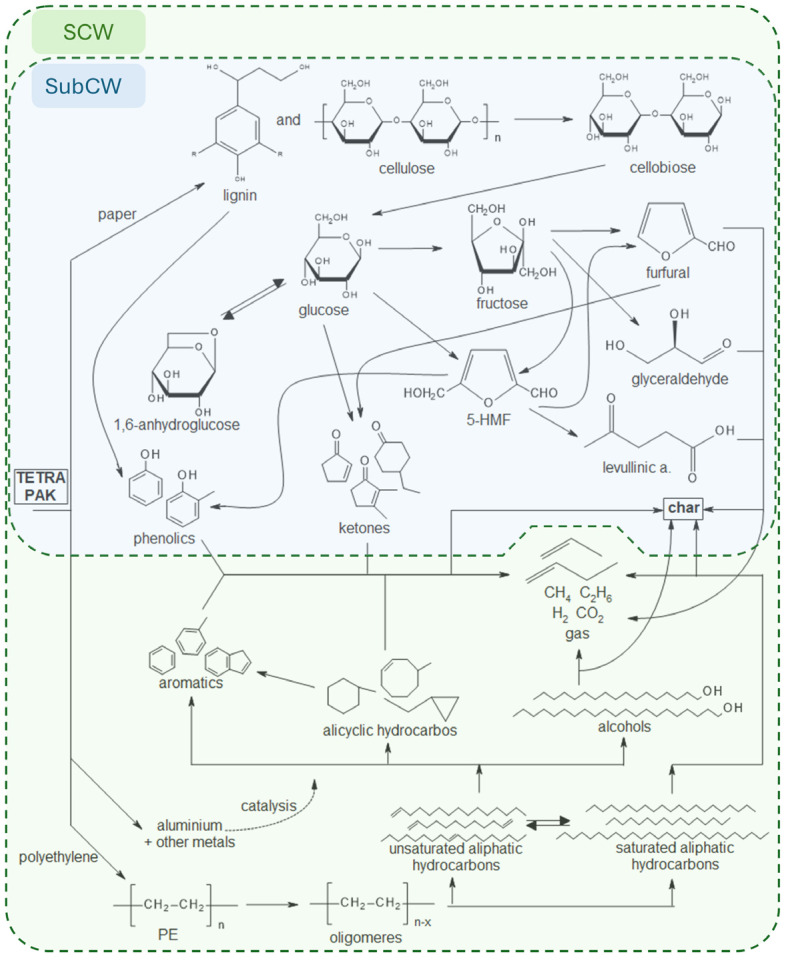
Possible waste tetra pak packaging degradation pathway in SubCW and SCW.

The possible degradation pathway of the waste tetra pak packaging in SCW was constructed based on the results of the components present in the oil and water phases. First, at SubCW conditions, the paper layer of the packaging was degraded, i.e., lignin to phenolic components (mainly 2-metoxyphenol) and cellulose to oligomers of cellobiose, which in turn were decomposed into monomeric glucose units [18]. The glucose was then converted to 1,6-anhydroglucose by a dehydration process or isomerized to fructose [36]. The fructose further decomposed into various derivatives, especially with increasing temperature, among which 1,6-anhydroglucose, glyceraldehyde, 5-HMF, furfural, and 5-MF were detected in the aqueous phase. 5-HMF is formed from glucose and fructose by dehydration. At higher temperatures and longer reaction times, 5-HMF then decomposed to levulinic acid and furfural or it was converted to phenolic components by ring opening and closing processes. Furfural and glucose can also form ketones directly by hydrogenation rearrangement [41]. In the PE layer, which started to decompose at a temperature higher than 425 °C (in SCW), the C–C bonds were first broken to form oligomers. These led to shorter saturated and unsaturated aliphatic hydrocarbons, which were converted into each other by hydrogenation and β-scission [23]. The unsaturated aliphatic hydrocarbons then formed alcohols by the process of hydration, alicyclic hydrocarbons by the process of cyclization, and aromatic hydrocarbons by the process of aromatization [42], which was especially noticed at longer rection times. With increasing reaction time, alicyclic hydrocarbons could be converted to aromatic hydrocarbons through the process of dehydrogenation. Cracking of the alkanes and alkenes at higher temperatures produced short, gaseous hydrocarbons. In the gas phase, unsaturated aliphatic hydrocarbons were converted to saturated aliphatic hydrocarbons by a hydrogenation reaction, and CO_2_ and H_2_ were formed by a steam reforming reaction [23]. As the reaction time increased, some char was also formed. Furthermore, the influence of catalysis by aluminium and other detected metals (Ni, Co, Cr) cannot be excluded in all the above mechanisms [43]. In view of all this, the degradation pathway shown in Figure 7 was constructed [16,18,23,36,41].

### 3.5. Comparison of One-Stage and Two-Stage Degradation Process

The results of this study showed some differences in the products obtained from waste tetra pak packaging when the degradation process was carried out in a single stage with SCW or in two stages with SubCW in the 1st stage and SCW in the 2nd stage. 

In single-stage hydrothermal degradation of waste tetra pak packaging, the highest *γ*(o) (60.7%) was obtained at 450 °C and a reaction time of 60 min. The overall *γ*(o) after two-stage hydrothermal degradation of packaging waste was higher than after single-stage degradation for the three different samples C-2 (1st stage 300 °C, 30 min; 2nd stage 450 °C, 15 min), D-1 (1st stage 300 °C, 60 min; 2nd stage 425 °C, 15 min), and D-2 (1st stage 300 °C, 60 min; 2nd stage 450 °C, 15 min). The maximum *γ*(o) in the two-stage decomposition was obtained for sample D-2 (65.5%), where the 1st stage was carried out at 300 °C and a reaction time of 60 min and the 2nd stage at 450 °C and a reaction time of 15 min.

The oils obtained using the single-stage process and the two-stage process also differed in their composition. When comparing the content of saturated and unsaturated aliphatic hydrocarbons in the oil obtained in the single-stage and the 2nd stage of two-stage degradation, it was found that in the single-stage degradation, the ratio of alkanes/alkenes in the oil is between 2.7 and 7.2 and decreases with increasing temperature and reaction time, while the ratio in the oil of the 2nd stage of two-stage degradation is between 1.1 and 3.8. A favorable interaction between cellulose and PE, which influences the formation of aliphatic hydrocarbons, has also been described in the literature [44]. Moreover, the ratio of aliphatic and aromatic compounds was lower in the oil of single-stage degradation (from 1.2 to 4.9) and increased with increasing temperature and reaction time as in the oil of two-stage degradation (2.2–13.6). The oil of the 2nd stage of two-stage degradation also contained a lower amount of ketones. This was a consequence of the fact that aromatic compounds and ketones were formed from the lignocellulosic material in the 1st stage of two-stage degradation and were removed with the oil of the 1st stage. 

The difference in the gas phase was mainly reflected in the amount of CO_2_, which was much higher in single-stage decomposition than in the 2nd stage of two-stage decomposition. CO_2_ was the dominant gas in SCW gasification of cellulose [45]. The composition of the gas phase obtained in a two-stage process, where most of the lignocellulosic material has been removed in the 1st stage, was similar to that of the gases produced during the decomposition of PE in SCW [23].

The TC value in the aqueous phase after single-stage degradation of tetra pak packaging waste was about half of the TC value after the 1st stage of two-stage degradation of tetra pak packaging waste. The TC value after single-stage degradation was slightly higher than the TC value after the 2nd stage of the two-stage degradation of waste tetra pak packaging. In the analysis of the aqueous phase, glucose, glyceraldehyde, levulinic acid, and furfurals were detected after the single-stage degradation. In the two-stage degradation of packaging waste, glucose, cellobiose, anhydro glucose, levulinic acid, and furfurals were detected after the 1st stage and glucose, fructose, levulinic acid, and furfurals after the 2nd stage. Glucose and levulinic acid are produced both in the single-stage degradation and in both stages of the two-stage degradation. The total content of glucose and levulinic acid was much higher in the two-stage degradation (approximately 9.9 times higher for glucose and 1.7 times higher for levulinic acid) than in the single-stage degradation, as further decomposition to furfurals already took place in single-stage degradation.

The composition of the solid residues obtained by the single-stage and two-stage processes was similar and consisted of carbon, aluminium, and other metals. The *γ*(s) for the one-stage degradation was between 11.4% and 13.9%, and the overall yield of the two-stage process was slightly higher between 13.8% and 18.3%, probably because of higher char formation in the two-stage degradation.

## 4. Conclusions

The degradation of waste tetra pak packaging in SubCW and SCW, a green and readily accessible rection medium, was successfully carried out. The experiments were carried out either in one stage (SCW) or in two stages (SubCW and SCW), investigating the influence of reaction temperature and time on the yield and composition of the resulting products. The products were obtained in the oil, aqueous, solid, and gas phases. The maximum yield of the oil phase was 60.7% for the one-stage process (450 °C, 60 min) and 65.5% for the two-stage process (1st stage: 300 °C, 60 min; 2nd stage: 450 °C, 15 min). The two-stage process has been proposed as a possible solution for stepwise recycling, effectively separating the different layers of the tetra pak packaging and extracting valuable components from the cardboard in the first stage (sugars, levulinic acid obtained in the water phase, and aromatic compounds and ketones in the oil phase) and from the plastic in the second stage (oil with the same composition as oil after SCW degradation of PE, which has a high potential for use as fuel). Higher temperatures and longer reaction times during SCW degradation resulted in more saturated aliphatic hydrocarbons in both the oil and gas phases. The gas phase, which was rich in ethane, propane, and butane, could be used as a fuel. The solid phase after completed degradation contained mainly aluminium, with traces of other metals from additives and dyes, and could be used as a raw material with further purification. This study shows that the degradation of waste tetra pak packaging with SubCW and SCW is a promising recycling method, whereby the desired end products can be influenced by the design of a one- or two-stage process.

## Figures and Tables

**Table 1:** Coding of samples obtained in two-stage degradation experiments: samples A to D obtained in the 1st stage, samples A-1 to D-1 and A-2 to D-2 obtained in the 2nd stage.

	1st Stage		2nd Stage	
Sample	T (°C)	t (min)	T (°C)	t (min)
A	250	30	/	/
B	250	60	/	/
C	300	30	/	/
D	300	60	/	/
A-1	250	30	425	15
B-1	250	60	425	15
C-1	300	30	425	15
D-1	300	60	425	15
A-2	250	30	450	15
B-2	250	60	450	15
C-2	300	30	450	15
D-2	300	60	450	15

**Table 2:** Proximate and ultimate analysis of waste tetra pak packaging.

**Proximate Analysis**	**wt.%**
Moisture	3.2
Ash	10.3
Volatile mater	83.6
Fixed carbon *	2.8
**Ultimate Analysis**	**wt.%**
C	48.0
H	8.2
N	0.1
S	0.3
O *	43.4

* Calculated by difference.

**Table 3:** Composition of gas phase after one-stage hydrothermal degradation of waste tetra pak packaging.

Degradation Conditions	425 °C, 15 min	425 °C, 60 min	450 °C, 15 min	450 °C, 60 min
Component	Peak Area (%)			
CO_2_	36.2	34.0	26.1	20.4
ethane	14.4	15.3	14.9	17.5
ethylene	1.4	1.4	2.9	1.4
propane	11.3	12.3	11.9	17.7
propene	7.9	6.9	10.0	7.2
butane	10.6	11.5	13.1	11.4
1-butene	3.8	4.2	4.9	4.7
pentane	4.4	4.8	5.1	6.2
1-pentene	1.6	1.6	2.1	2.0
Z-2-pentene	0.0	1.9	2.5	1.8
hexane	1.3	4.4	3.6	4.1
E-2-hexene	0.5	0.5	0.8	0.5
other	6.6	1.1	2.3	5.1

**Table 4:** Composition of gas phase after 2nd stage of two-stage hydrothermal degradation of waste tetra pak packaging.

Sample	A-1	A-2	B-1	B-2	C-1	C-2	D-1	D-2
Component	Peak Area (%)							
CO_2_	15.5	12.4	9.8	7.4	4.6	2.6	1.0	0.3
ethane	14.2	18.3	11.6	16.9	8.9	15.7	7.2	14.6
ethylene	3.2	3.4	3.0	2.8	2.6	2.0	2.1	1.7
propene	14.0	14.9	13.1	13.8	12.0	12.1	10.3	11.2
propane	11.6	13.8	12.9	16.2	14.9	19.7	16.6	22.8
2-methyl-1-propene	8.4	7.2	7.9	5.8	7.1	4.2	6.3	3.3
butane	4.2	5.0	7.9	8.2	10.3	11.6	15.8	14.0
1-butene	7.0	6.8	10.2	7.8	11.9	8.5	12.6	9.6
pentane	7.9	4.8	8.2	5.5	8.2	6.3	8.3	6.8
1-pentene	2.8	3.0	2.8	2.6	3.1	2.8	3.2	3.0
Z-2-pentene	2.4	1.0	2.5	1.4	2.8	1.5	2.9	1.8
hexane	2.6	3.0	3.0	3.5	4.2	4.3	5.5	4.8
E-2-hexane	0.9	1.1	2.0	2.2	3.5	3.3	4.6	3.6
other	5.5	5.3	5.1	5.9	5.9	5.4	3.6	2.5

## Data Availability

Data are contained within the article or Supplementary Material.

## Supplementary Materials

The following supporting information can be downloaded at: https://www.mdpi.com/article/10.3390/polym16131879/s1; Figure S1: EDS image of solid phase obtained after one-stage hydrothermal degradation of waste tetra pak packaging at 450 °C and reaction time of 60 min; Figure S2: FTIR spectra of solid phase after two-stage hydrothermal degradation of waste tetra pak packaging; (a) 1st stage, (b) 2nd stage; Figure S3: EDS image of solid phase obtained after second stage of two-stage hydrothermal degradation of waste tetra pak packaging; (a) sample D-1, (b) sample D-2; Table S1: Most represented compounds in oil phase after one-stage hydrothermal degradation of waste tetra pak packaging; Table S2: Elemental composition by weight of solid phase obtained after one-stage hydrothermal degradation of waste tetra pak packaging at 450 °C and reaction time of 60 min; Table S3: TC concentration, sugars, and their derivates in aqueous phase after one-stage hydrothermal degradation of waste tetra pak packaging; Table S4: Most represented compounds in oil phase after first stage in two-stage hydrothermal degradation of waste tetra pak packaging; Table S5: Most represented compounds in oil phase after second stage in two-stage hydrothermal degradation of waste tetra pak packaging; Table S6: Sugars and their derivates in aqueous phase after 1st and 2nd stage of two-stage hydrothermal degradation of waste tetra pak packaging; Table S7: Elemental composition by weight of solid phase obtained after second stage of two-stage hydrothermal degradation of waste tetra pak packaging.
