# Supplementary material for: Degradation of Waste Tetra Pak Packaging with Hydrothermal Treatment in Sub-/Supercritical Water
Source: Polymers (Basel). 2024 Jul 1;16(13):1879. doi: 10.3390/polym16131879 (PMC11243872; doi:10.3390/polym16131879)
Supplement: Supplementary file 1 [file polymers-16-01879-s001.zip › polymers-3007976-supplementary.pdf]

Supplementary Material

Degradation of waste tetra pak packaging with hydrothermal treatment in sub-/supercritical water

Mihael Irgolič[a], Maja Čolnik[a], Petra Kotnik[a,b], Mojca Škerget[a,*]

[a] University of Maribor, Faculty of Chemistry and Chemical Engineering, Smetanova ulica 17, SI-2000 Maribor, Slovenia

[b] University of Maribor, Faculty of Medicine, Taborska ulica 8, SI-2000 Maribor, Slovenia

*mojca.skerget@um.si

1. One-stage degradation

Table S1. Most represented compounds in oil phase after one-stage hydrothermal degradation of waste tetra pak packaging.

		**Reaction conditions**	**425 °C 15 min**	**425 °C 60 min**	**450 °C 15 min**	**450 °C 60 min**
Ret. time (min)	CAS #	Compound names*	Area of peak (%)			
		**Saturated aliphatic hydrocarbons (total)**	**29.00**	**34.21**	**32.37**	**46.33**
4.924	110-54-3	Hexane	/	0.15	0.06	0.12
6.885	111-65-9	Octane	/	0.17	0.55	0.22
7.221	111-84-2	Nonane	0.10	0.69	0.94	0.14
10.021	1120-21-4	Undecane	/	1.21	1.61	2.28
10.704	112-40-3	Dodecane	2.10	2.18	/	/
10.698	629-50-5	Tridecane	/	/	2.93	3.74
13.464	629-59-4	Tetradecane	2.42	/	2.46	3.42
13.525	629-62-9	Pentadecane	1.84	2.02	/	6.61
14.729	544-76-3	Hexadecane	2.67	5.11	2.12	0.50
15.970	629-92-5	Nonadecane	3.19	3.09	/	3.74
16.729	112-95-8	Eicosane	2.52	1.51	2.57	9.12
17.296	629-94-7	Heneicosane	6.19	15.55	6.97	12.76
20.214	630-01-3	Hexacosane	2.60	3.53	5.49	3.65
24.932	630-02-4	Octacosane	5.32	/	2.44	/
		**Unsaturated aliphatic hydrocarbons (total)**	**4.05**	**6.95**	**5.23**	**17.15**
6.253	592-76-7	1-Heptene	/	/	0.33	0.62
7.010	124-11-8	1-Nonene	/	/	0.58	0.98
7.410	872-05-9	1-Decene	/	/	1.44	/
10.536	112-41-4	1-Dodecene	/	/	/	2.24
13.321	1120-36-1	1-Tetradecene	/	/	/	2.54
14.595	13360-61-7	1-Pentadecene	/	/	/	2.79
15.237	6765-39-5	1-Heptadecene	/	3.60	/	4.85
19.605	7206-25-9	9-Octadecene, (E)-	2.86	3.35	2.88	/
23.203	27519-02-4	9-Tricosene, (Z)-	/	/	/	2.25
		**Alicyclic hydrocarbons (total)**	**2.04**	**2.82**	**4.55**	**/**
9.010	74663-85-7	Cyclopropane, nonyl-	2.04	2.82	3.51	/
6.250	61142-41-4	Cyclooctane, ethenyl-	/	/	1.03	/
		**Aromatic hydrocarbons (total)**	**28.37**	**21.94**	**18.20**	**13.06**
5.149	100-41-4	Ethylbenzene	/	/	2.32	1.01
6.909	108-95-2	Phenol	4.56	1.74	1.53	0.98
8.074	95-48-7	o-Cresol	1.87	1.60	1.98	1.37
8.400	108-39-4	m-Cresol	3.77	3.29	2.14	1.29
8.414	106-44-5	p-Cresol	1.21	2.01	1.38	2.07
9.115	90-00-6	2-Ethylphenol	0.52	/	/	0.68

9.375	620-17-7	3-Ethylphenol	1.36	0.87	/	/
9.529	105-67-9	2,4-Xylenol	/	1.96	1.63	/
9.965	526-75-0	2,3-Xylenol	/	3.20	/	0.91
10.187	95-65-8	3,4-Xylenol	/	3.20	/	1.22
10.213	120-80-9	Catechol	4.00	/	1.39	/
11.111	452-86-8	4-Methylcatechol	3.51	/	/	0.86
12.230	1124-39-6	4-Ethylcatechol	1.55	/	1.90	/
		Polycyclic aromatics (PAH)	*3.54*	*2.08*	*1.07*	*0.88*
		**Alcohols (total)**	**11.71**	**14.47**	**16.90**	**16.20**
11.971	112-53-8	1-Dodecanol	1.01	1.58	3.07	2.50
18.572	15594-90-8	1-Heneicosanol	1.79	5.66	6.15	/
20.068	1454-85-9	n-Heptadecanol-1	1.09	1.86	1.27	/
20.700	1454-84-8	n-Nonadecanol-1	1.63	/	1.50	5.15
23.199	661-19-8	1-Docosanol	3.15	5.37	3.27	2.45
24.789	506-51-4	n-Tetracosanol-1	1.69	/	/	2.29
28.659	2004-39-9	1-Heptacosanol	1.36	/	/	5.15
		**Ketones (total)**	**9.55**	**12.35**	**15.21**	**1.28**
4.025	120-92-3	Cyclopentanone	/	6.77	/	/
4.698	1120-72-5	Cyclopentanone, 2-methyl-	/	2.22	2.18	/
4.757	1757-42-2	Cyclopentanone, 3-methyl-	/	/	1.22	/
6.535	2758-18-1	2-Cyclopenten-1-one, 3-methyl-	1.10	/	1.16	/
7.783	1121-05-7	2-Cyclopenten-1-one, 2,3-dimethyl-	3.15	2.22	3.16	1.28
9.245	5682-69-9	2-Cyclopenten-1-one, 3-ethyl-	1.27	/	2.7	/
		**Other (total)**	**15.27**	**7.26**	**7.55**	**5.99**

*Similarity with NIST library more than88 %.

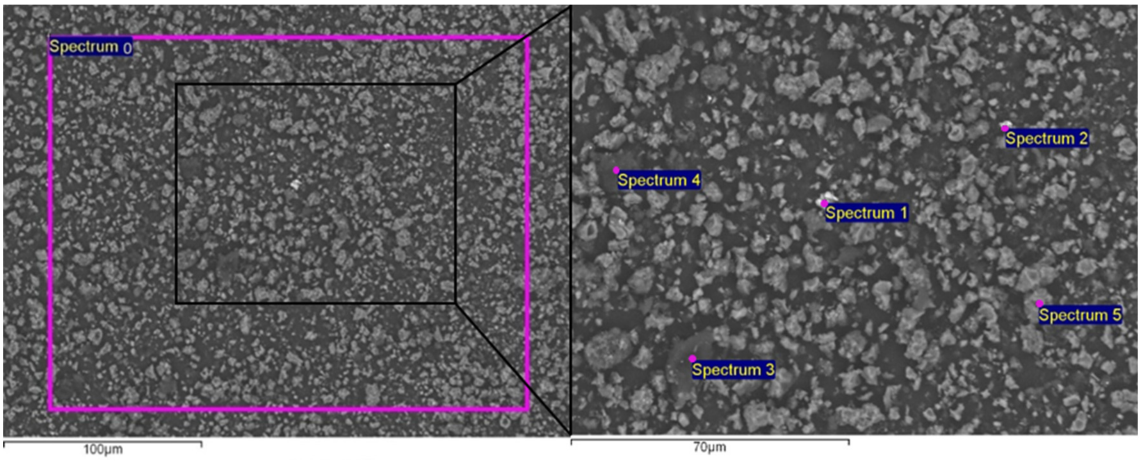

Figure S1. EDS image of solid phase obtained after one-stage hydrothermal degradation of waste tetra pak packaging at 450 °C and reaction time of 60 min.

Table S2. Elemental composition by weight of solid phase obtained after one-stage hydrothermal degradation of waste tetra pak packaging at 450 °C and reaction time of 60 min.

Spectrum/ Element	C	O	Al	Si	S	Ca	Cr	Fe	Co	Ni
	wt. %									
**Spectrum 0**	/	59.79	31.53	1.54	/	7.13	/	/	/	/
**Spectrum 1**	34.28	14.53	1.69	/	11.52	0.70	5.58	2.37	/	29.33
**Spectrum 2**	57.51	11.66	1.36	0.28	/	0.36	/	5.38	4.09	19.37
**Spectrum 3**	88.23	9.66	1.29	0.46	/	0.37	/	/	/	/
**Spectrum 4**	86.20	8.81	3.43	0.57	/	0.99	/	/	/	/
**Spectrum 5**	17.62	41.04	38.48	1.08	/	1.77	/	/	/	/

Table S3. Total carbon (TC) concentration, sugars, and their derivates in aqueous phase after one-stage hydrothermal degradation of waste tetra pak packaging.

degradation conditions	TC (mg/L)	c_{glucose} (mg/mL)	$c_{\text{glyceraldehyde}}$ (mg/mL)	$c_{\text{levulinic a.}}$ (mg/mL)	$c_{\text{5-HMF}}$ (mg/mL)	c_{furfural} (mg/mL)
**425 °C, 15 min**	3792	0.046	0.077	1.714	/	0.006
**425 °C, 60 min**	3306	0.002	0.072	1.289	/	0.007
**450 °C, 15 min**	3838	0.037	0.066	1.440	0.008	0.003
**450 °C, 60 min**	2466	0.003	0.063	1.327	0.002	0.010

3. Two-stage degradation

Table S4. Most represented compounds in oil phase after first stage in two-stage hydrothermal degradation of waste tetra pak packaging.

		**Sample**	**A**	**B**	**C**	**D**
Ret. time (min)	CAS #	Compound names*	Area of peak (%)			
		**Aromatic hydrocarbons (total)**	**27.64**	**45.18**	**27.09**	**33.67**
4.682	534-22-5	Furan, 2-methyl-	/	5.47	/	/
8.074	95-48-7	o-Cresol	5.37	4.00	3.04	2.93
8.400	108-39-4	m-Cresol	2.66	2.75	2.45	2.12
8.585	90-05-1	2-mathoxyphenol	19.61	32.96	21.60	28.62
		**Ketones (total)**	**42.61**	**25.55**	**37.29**	**38.92**
4.622	930-30-3	2-Cyclopenten-1-one	28.40	25.55	12.43	21.78
5.689	765-69-5	1,2-Cyclopentanedione, 2-methyl-	/	/	24.86	9.24
7.546	765-70-8	1,2-Cyclopentanedione, 3-methyl-	14.21	/	/	7.90
		**Other (total)**	**29.75**	**29.17**	**35.62**	**27.51**
23.874	112-91-4	Oleanitrile	9.33	8.52	13.14	5.81

*Similarity with NIST library more than 88%.

Table S5. Most represented compounds in oil phase after second stage in two-stage hydrothermal degradation of waste tetra pak packaging.

		**Sample**	**A-1**	**A-2**	**B-1**	**B-2**	**C-1**	**C-2**	**D-1**	**D-2**
Ret. time (min)	CAS #	Compound names*	Area of peak (%)							
		**Saturated aliphatic hydrocarbons (total)**	**29.20**	**39.33**	**29.88**	**19.87**	**32.66**	**41.50**	**37.46**	**36.59**
4.924	110-54-3	Hexane	/	0.11	/	/	/	0.27	0.12	0.62
6.885	111-65-9	Octane	/	0.26	/	/	/	0.22	0.06	0.53
7.221	111-84-2	Nonane	0.96	0.85	/	/	/	0.47	0.46	0.21
10.021	1120-21-4	Undecane	/	3.66	1.91	2.99	1.6	4.21	1.3	4.69
10.704	112-40-3	Dodecane	2.64	2.15	2.25	2.4	2.28	2.43	1.99	2.22
13.464	1120-36-1	Tetradecane	2.28	/	/	/	/	2.72	1.91	/
13.525	629-62-9	Pentadecane	2.02	2.14	/	3.02	1.19	2.46	2	/
14.727	54833-48-6	Heptadecane, 2,6,10,15-tetramethyl-	5.49	5.98	/	3.91	/	/	/	2.93
15.970	629-92-5	Nonadecane	/	/	/	/	1.27	2.93	3.56	/
16.729	112-95-8	Eicosane	/	/	2.67	/	2.72	8.85	3.24	2.5
17.296	629-94-7	Heneicosane	/	/	/	/	12.49	11.26	12.07	/
20.214	630-01-3	Hexacosane	3.34	9.22	15.64	/	4.05	/	3.35	13.95
21.922	1561-02-0	2-Methylhexacosane	6.46	6.9	4.08	/	5.49	/	4.17	/
24.932	630-02-4	Octacosane	/	/	3.33	3.61	/	/	/	/
28.862	7098-22-8	Tetratetracontane	/	6.8	/	3.95	1.58	5.66	3.34	6.89
		**Unsaturated aliphatic hydrocarbons (total)**	**9.25**	**11.17**	**7.88**	**17.66**	**14.59**	**19.60**	**15.67**	**12.38**
6.253	592-76-7	1-Heptene	/	/	0.13	0.84	0.16	0.79	0.24	0.83
7.010	124-11-8	1-Nonene	/	/	0.58	0.92	0.49	0.88	0.95	0.92
7.410	872-05-9	1-Decene	/	/	/	2.19	1.2	2.04	/	1.77
10.236	41446-66-6	5-Tetradecene, (E)-	/	/	/	2.14	/	0.25	/	/
13.320	2437-56-1	1-Tridecene	1.89	/	/	/	2.59	2.8	2.13	/
13.619	10374-74-0	7-Tetradecene	/	0.51	0.76	3.48	/	/	/	/
14.595	13360-61-7	1-Pentadecene	/	/	/	/	/	/	2.97	/
15.237	6765-39-5	1-Heptadecene	1.83	/	/	/	1.9	3.37	/	/

15.620	18435-45-5	1-Nonadecene	/	0.51	/	/	/	/	/	/
15.863	42448-90-8	9-Eicosene, (E)-	5.54	2.75	0.81	/	2.63	3.12	2.61	2.52
16.136	74685-30-6	5-Eicosene, (E)-	/	0.93	0.69	/	/	/	/	1.75
17.020	21964-51-2	1,15-Hexadecadiene	/	0.69	/	/	/	/	/	/
19.605	7206-25-9	9-Octadecene, (E)-	/	5.78	2.88	2.32	4.51	6.34	5.03	4.53
		**Alicyclic hydrocarbons (total)**	**2.71**	**5.54**	**1.41**	**1.80**	**3.42**	**5.01**	**4.05**	**3.61**
7.439	74663-86-8	Cyclopropane, 1-ethyl-2-heptyl-	/	2.00	/	/	/	/	/	/
9.010	74663-85-7	Cyclopropane, nonyl-	1.03	3.54	0.76	0.96	2.41	5.01	3.15	3.09
9.365	81983-71-3	Cyclohexane, 1,1-dimethyl-2-propyl-	/	/	0.65	0.84	1.01	/	0.91	0.53
		**Aromatic hydrocarbons (total)**	**16.88**	**3.71**	**9.47**	**3.44**	**14.82**	**9.10**	**17.27**	**7.09**
6.909	108-95-2	Phenol	3.75	/	0.88	/	1.7	0.66	1.95	0.57
8.074	95-48-7	o-Cresol	1.57	0.59	0.83	0.86	0.78	0.82	0.81	0.47
8.400	108-39-4	m-Cresol	2.45	0.97	1.64	0.68	1.64	1.53	1.97	1.19
9.529	105-67-9	2,4-Xylenol	1.39	0.61	/	0.59	0.90	/	1.10	0.63
9.965	526-75-0	2,3-Xylenol	/	/	/	/	/	0.40	0.37	/
10.187	95-65-8	3,4-Xylenol	/	/	/	0.59	/	0.38	/	0.98
10.213	120-80-9	Catechol	/	/	0.93	/	/	0.65	/	/
11.111	452-86-8	4-Methylcatechol	1.25	0.44	1.65	0.48	1.01	0.64	1.13	/
12.230	1124-39-6	4-Ethylcatechol	1.15	/	/	/	0.98	/	0.85	/
		Polycyclic aromatics (PAH)	*2.20*	*0.48*	*1.62*	/	*3.22*	*1.84*	*3.74*	*1.57*
		**Alcohols (total)**	**2.35**	**31.50**	**19.98**	**22.69**	**12.04**	**24.18**	**20.82**	**27.82**
11.971	112-53-8	1-Dodecanol	2.35	2.37	1.92	/	/	/	/	2.03
15.829	112-70-9	n-Tridecan-1-ol	/	0.46	2.04	2.38	/	/	/	/
18.572	15594-90-8	1-Heneicosanol	/	7.96	2.74	2.6	2.42	6.85	2.92	8.85
20.068	1454-85-9	n-Heptadecanol-1	/	4.05	7.11	4.71	4.57	3.56	5.79	5.81
20.070	1454-84-8	n-Nonadecanol-1	/	/	/	1.82	/	/	/	/
23.199	661-19-8	1-Docosanol	/	7.6	1.7	7.39	2.16	3.23	2.75	3.43
24.789	506-51-4	n-Tetracosanol-1	/	/	3.62	/	/	/	2.9	/
28.659	2004-39-9	1-Heptacosanol	/	6.81	/	/	2.59	9.58	5.6	7.69
		**Ketones (total)**	**6.43**	**1.28**	**2.33**	**/**	**4.64**	**/**	**2.38**	**1.07**

5.689	765-69-5	1,2-Cyclopentanedione, 2-methyl-	3.00	0.55	/	/	/	/	/	/
7.546	765-70-8	1,2-Cyclopentanedione, 3-methyl-	1.21	/	/	/	/	/	/	/
7.783	3883-58-7	1,2-Cyclopentanedione, 2,3-dimethyl-	2.22	0.73	2.33	/	4.64	/	2.38	1.07
		**Other (total)**	**33.16**	**7.46**	**29.05**	**34.45**	**18.07**	**0.62**	**1.91**	**11.44**
15.917	1000382-54-3	Carbonic acid, tridecyl vinyl ester	/	/	/	4.24	/	/	/	/
20.055	1000382-54-4	Carbonic acid, octadecyl vinyl ester	9.22	/	4.56	/	/	/	/	/
23.339	1000382-54-5	Carbonic acid, tetradecyl vinyl ester	3.00	/	8.48	6.87	5.49	/	/	3.63
26.675	2243791-78-6	Carbonic acid, eicosyl vinyl ester	7.64	3.07	10.06	18.26	9.81	/	/	7.19

*Similarity with NIST library more than 88%.

Table S6. Sugars and their derivates in aqueous phase after 1st and 2nd stage of two-stage hydrothermal degradation of waste tetra pak packaging.

Sample	c_{glucose} (mg/mL)	c_{fructose} (mg/mL)	$c_{\text{cellobiose}}$ (mg/mL)	$c_{\text{anh. glucose}}$ (mg/mL)	$c_{\text{levulinic a.}}$ (mg/mL)	$c_{\text{5-HMF}}$ (mg/mL)	c_{furfural} (mg/mL)	$c_{\text{5-MF}}$ (mg/mL)
**A**	0.093	/	0.103	3.057	1.742	0.064	/	/
**B**	0.135	/	0.013	2.108	2.171	0.078	0.005	/
**C**	0.246	/	0.211	4.925	2.149	/	0.006	0.004
**D**	0.286	/	0.080	5.410	2.498	/	/	0.005
**A-1**	0.003	0.060	/	/	0.751	0.008	/	/
**A-2**	0.007	0.063	/	/	0.662	0.013	/	/
**B-1**	0.002	0.016	/	/	0.593	/	0.004	/
**B-2**	0.039	0.017	/	/	0.682	/	0.007	/
**C-1**	0.041	0.293	/	/	0.405	/	0.005	/
**C-2**	0.114	0.042	/	/	0.385	/	0.007	/
**D-1**	0.042	0.283	/	/	0.421	/	0.008	0.004
**D-2**	0.169	0.036	/	/	0.385	/	0.010	0.006

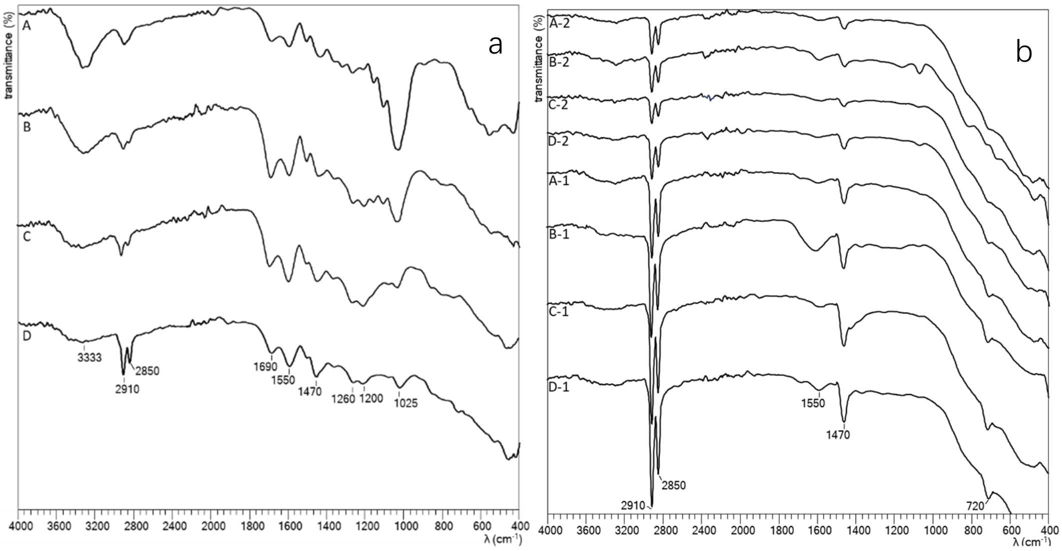

Figure S2. FTIR spectra of solid phase after two-stage hydrothermal degradation of waste tetra pak packaging. (a) 1st stage, (b) 2nd stage.

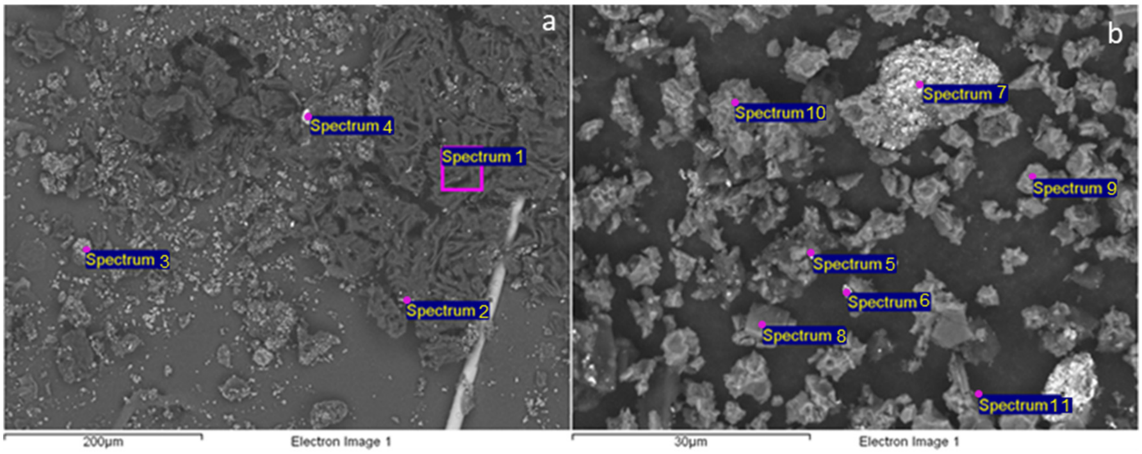

Figure S3. EDS image of solid phase obtained after second stage of two-stage hydrothermal degradation of waste tetra pak packaging. (a) sample D-1, (b) sample D-2.

Table S7. Elemental composition by weight of solid phase obtained after second stage of two-stage hydrothermal degradation of waste tetra pak packaging.

**Spectrum/ Element**	C	O	Al	Si	S	Ca	Cr	Mg	Mo	Ni
	wt. %									
**Spectrum 1**	97.09	2.91	/	/	/	/	/	/	/	/
**Spectrum 2**	73.02	17.90	1.60	5.09	/	2.18	/	0.21	/	/
**Spectrum 3**	21.31	45.21	31.27	0.54	/	0.77	/	/	/	/
**Spectrum 4**	86.46	1.93	1.36	0.57	3.51	/	1.92	/	/	4.81
**Spectrum 5**	27.80	2.32	1.45	/	15.27	/	/	/	/	53.16
**Spectrum 6**	33.24	10.69	5.67	/	10.61	/	/	/	/	39.80
**Spectrum 7**	26.93	29,10	3.00	/	0.55	/	0.98	23.95	23.95	15.49
**Spectrum 8**	17.59	17.38	65.03	/	/	/	/	/	/	/
**Spectrum 9**	3.48	49.84	45.58	/	/	1.09	/	/	/	/
**Spectrum 10**	6.52	50.74	42.73	/	/	/	/	/	/	/
**Spectrum 11**	37.17	12.29	2.09	/	17.03	/	0.73	/	/	30.69
